# Drug survival of systemic immunosuppressive treatments for atopic dermatitis in a long-term pediatric cohort

**DOI:** 10.1016/j.ijwd.2021.07.005

**Published:** 2021-07-22

**Authors:** Stine Elsgaard, Anna Kathrine Danielsen, Jacob P. Thyssen, Mette Deleuran, Christian Vestergaard

**Affiliations:** a Department of Dermatology, Aarhus University Hospital, Aarhus Denmark; b Department of Dermatology, Bispebjerg Hospital, University of Copenhagen, Denmark

**Keywords:** Atopic dermatitis, pediatric, systemic immunosuppressive treatment, methotrexate, azathioprine, drug survival

## Abstract

**Background:**

*:* Systemic immunosuppressive treatments are central in the treatment of severe atopic dermatitis (AD). Yet, comparative data are sparse on the performance of such immunosuppressive treatments in pediatric cohorts with severe AD.

**Objective:**

*:* This study aimed to examine the drug survival of systemic immunosuppressive treatments in a cohort of children with severe AD.

**Methods:**

*:* A retrospective pediatric cohort was identified using diagnosis and treatment codes registered in medical charts. In total, 135 cases were identified; of these, 36 were excluded. All information was obtained through examination of clinical records. Drug survival was analyzed with Kaplan–Meier plots, and a log-rank test was used to test for differences in drug survival.

**Results:**

*:* First-line treatment was primarily methotrexate (MTX; n = 63) and azathioprine (AZA; n = 32). For MTX, the drug survival rates were 69%, 50%, and 18% after 1, 2, and 4 years, respectively, with a median drug survival time of 1.58 years. For AZA, these rates were 63%, 53%, and 21%, respectively, with a median drug survival time of 1.14 years. There was no significant difference in drug survival between the treatments. The main reason for discontinuation was adverse effects (MTX: 25%; AZA: 41%). Despite this, a majority of patients experienced a good effect at the moment of discontinuation or data-lock (MTX: 60%; AZA: 53%), and treatment effect assessed as improvement in sleep quality was highly significant (*p* = .001). Second-line treatments included MTX (n = 12), AZA (n = 7), and cyclosporine (n = 5). These showed a median drug survival time of 1.8, 0.2, and 0.885 years, respectively.

**Conclusion:**

*:* MTX and AZA were the dominant first-line treatments prescribed and were safe and equally valuable treatment options for severe childhood AD with similar drug survival outcomes. MTX was the most used second-line treatment.

**What is known about this subject in regard to women and their families?**

• Atopic dermatitis is a chronic skin disorder associated with profound discomfort, potentially affecting patients both physically and psychologically.
• Immunosuppressive treatments, including methotrexate and azathioprine, are frequently used in treating severe atopic dermatitis in children.
• There is very little knowledge on the drug survival of these drugs in children, and very little is known about the efficacy and side effects in this population.

**What is new from this article as messages for women and their families?**

• Methotrexate and azathioprine are safe and effective in treating severe atopic dermatitis in children, although there is a high incidence of side effects leading to discontinuation of the treatments.
• These treatments had similar drug survival outcomes and are equally valuable.
• Sleep quality proved to be an important measurable marker for the effect of the treatments.

Alt-text: Unlabelled box

## Introduction

Atopic dermatitis (AD) is a chronic, relapsing, inflammatory, pruritic, and eczematous skin disease (Weidinger and Novak, 2016). AD affects up to 20% of the pediatric population in developed countries, and although the disease can occur at any age, in 60% of cases it manifests during the first year of life (Weidinger and Novak, 2016). The major pathophysiological features contributing to disease development are defects in the epidermal barrier function and inflammation in the skin dominated by a T-helper 2/T-helper 22 lymphocyte–skewed cytokine production (Weidinger and Novak, 2016). AD is associated with infectious and allergic comorbidities and causes substantial psychological morbidity (Thyssen et al., 2020). The impaired skin barrier contributes to greater exposure of potential allergens, which ultimately can cause type 1 and type 4 hypersensitivity reactions, such as food allergy, allergic rhinitis, asthma, and contact allergy (Brunner et al., 2017; Flohr et al., 2014; Silverberg, 2019; Simonsen et al., 2018).

The pruritus and skin lesions associated with AD can affect sleep, physical and mental development, self-esteem, social interactions, and participation in school, posing a potential risk of psychological affects as well (Cubero et al., 2016). Moreover, several studies have found an association between AD and attention deficit/hyperactivity disorder and depression (Riis et al., 2016; Rønnstad et al., 2018; Strom et al., 2016). The risk of these comorbidities likely increases with the severity of the disease; thus, the management of severe AD should be of high priority.

The aim of the treatments for AD is to improve symptoms and achieve long-term disease control through restoration of the epidermal barrier by use of emollients and through reduction of the inflammatory process in the skin (Vestergaard et al., 2020; Wollenberg et al., 2016; 2018a; 2018b). Topical corticosteroids and/or topical calcineurin inhibitors are first-line therapies, whereas systemic glucocorticoids are used only as short-term therapy for severe disease flares and should be used reservedly (Proudfoot et al. 2013; Wollenberg et al., 2018a). In severe chronic cases with AD refractory to topical therapy and wet wrap therapy, systemic treatment may be used also in children (Weidinger and Novak, 2016; Wollenberg et al., 2016). The only systemic immunosuppressant licensed for AD in Europe is cyclosporine (CsA), but azathioprine (AZA), methotrexate (MTX), and mycophenolate mofetil (MMF) are commonly used off label (Proudfoot et al., 2013). Dupilumab (anti IL-4/IL-13 receptor) has recently been licensed for children from the age of 6 years. In the United Kingdom and Denmark, AZA was the preferred first-line choice until recently (Proudfoot et al., 2013). The treatment of children with systemic immunosuppressive agents poses a particular challenge. Consideration must be given to how treatment can affect the child's well-being, development, and growth.

Drug survival analysis is a standard method used to describe daily practice treatment results. Prior work has documented the drug survival of several immunosuppressive treatments in patients with AD, as well as the efficacy of these treatments. Efficacy has been examined in both adult and pediatric patients; however, to date, a drug survival analysis has only been conducted on adult patients (Gerbens et al., 2018; Law Ping Man et al., 2018; Politiek et al., 2016; van der Schaft et al., 2015; 2016). This knowledge gap deserves special attention to advance the understanding and ensure optimal treatment of severe childhood AD.

## Methods

### Study design

This study was designed as a retrospective follow-up study among children with AD who were treated with at least one of the four systemic immunosuppressive drugs (CsA, AZA, MTX, and MMF) between 2011 and 2020 at the Department of Dermatology, Aarhus University Hospital, Denmark. Data-lockdown was set for March 1, 2020. Data on patient demographics and treatment characteristics were obtained by review of the clinical records. The study was approved by the Danish Patient Safety Authority (J.No. 3-3013-3287/1) and the Danish Data Protection Agency (J.No. 1-16-02-382-19).

### Patient selection

The pediatric cohort of patients with AD was identified based on diagnostic codes (International Classification of Diseases, 10th Revision, Clinical Modification: L20.x) and treatment codes registered in the medical records. The codes included BLHM3 (MTX orally and AZA), BWHA115 (MTX inj.), BOHJ22A (MMF), BOHJ10 (Immunoglobulin), BOHJ20 (CsA), and BOHJ18B8 (Dupilumab). The search included patients treated in the Department of Dermatology, Aarhus University Hospital, who were age <18 years at the time of treatment initiation.

### Variables

The following general patient information was obtained: age/birth date, gender, age at time of AD onset, family history of atopy, history of allergies, use of prednisolone, and strongest topical corticosteroid ever used (groups I-IV). Where age at AD onset was described in the charts with the terms “since infancy” or “since childhood,” the age was set to 6 months and 2 years, respectively.

Concerning treatment, the following variables were obtained: dates of initiation and discontinuation, starting dose and maximum dose during treatment, reason for discontinuation, treatment pauses, sleep problems before and during treatment, and the effect of the treatment. Treatment discontinuation was considered to be an interruption of treatment for at least 3 months. Reasons for discontinuation included 1) adverse effects (counting both sickness and biochemical alterations), 2) inefficacy (defined as inadequate/unsatisfying improvement of AD), 3) controlled AD, and 4) unknown (i.e., either unmentioned or due to other reasons, e.g., problems with administration or own initiative). The treatment effect was rated as good, moderate, or none/insufficient based on the doctor's assessments noted in the charts. Where information was inadequate, the effect was rated as unknown.

### Drug survival and statistical analysis

Drug survival was analyzed with the Kaplan–Meier method with regard to overall discontinuation. For each treatment, survival curves were analyzed separately, depending on the treatment line. Patients still using the treatment at the end of the study were censored. Differences in drug survival for each drug in first-line treatment were analyzed using the log-rank test. Predictive factors (age at treatment initiation, gender, and prescribed drug) were analyzed using univariate cox regressions. Pearson's χ^2^ test was performed to test for differences in demography of the patients, treatment effect, and reasons for discontinuation. In second- to fourth-line treatments, the sample sizes were too small (n < 30) to allow for proper statistical evaluations; thus, no χ^2^ test was performed on these groups. Statistical analysis was performed using STATA/IC 16.0 (for the survival analysis, log-rank test, and Cox regression) and R software, version 3.6.3 (for qualitative and quantitative data processing, χ^2^ test, and Fisher's exact test). Measures of central tendency and dispersion are shown as mean ± standard deviation (SD) unless otherwise indicated.

## Results

### Study cohort

**Fig. 1:**
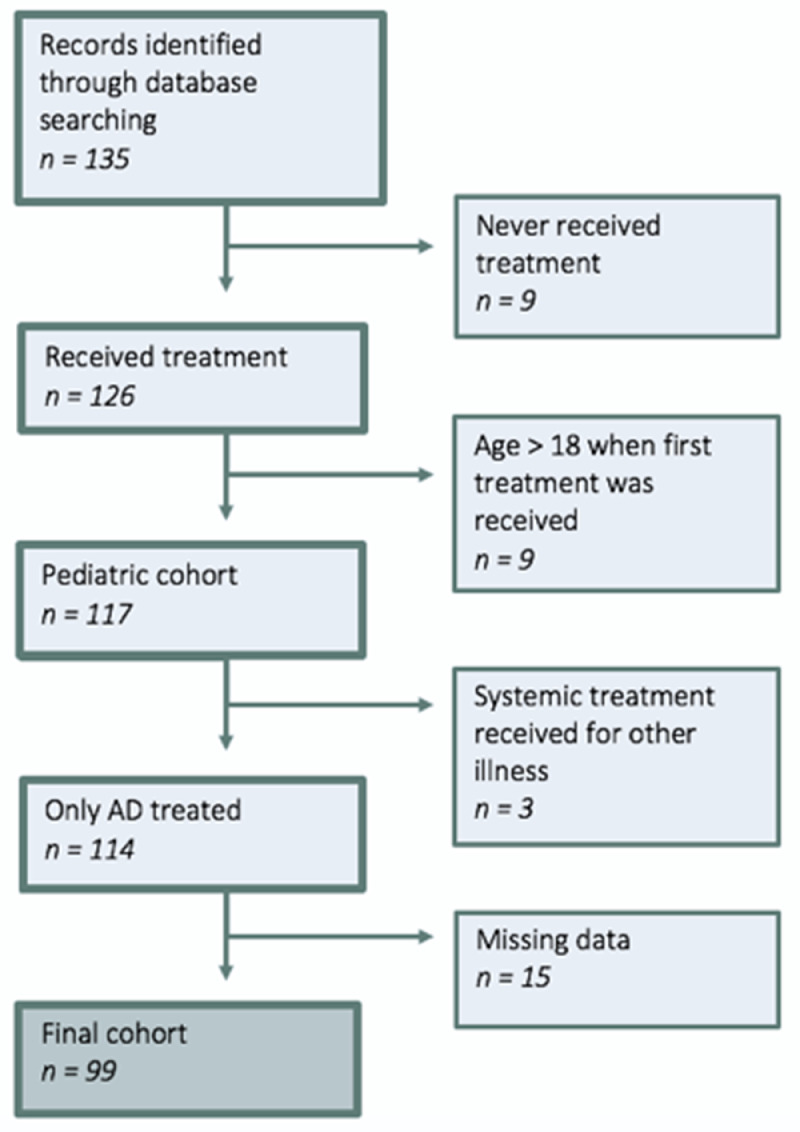
Flow diagram of the process of exclusion leading to a cohort of 99 eligible cases from a total of 135 identified records.

A total of 135 children were identified. All 135 patients’ medical records were reviewed and, during this process, a total of 36 patients were excluded. These cases were excluded for the following reasons: 1) error in the database treatment codes, meaning that some of the patients (n = 9) had not received systemic treatment; 2) the patient was age >18 years when systemic treatment was initiated (n = 9); 3) the patient received systemic treatment because of other illnesses, such as arthritis (n = 3); and 4) lack of information/data in the medical records (n = 15). The process of exclusion is shown in Figure 1.

### Patient and treatment characteristics

In total, 99 patients were included in the statistical analyses. The baseline characteristics are shown in Table 1. The cohort's mean age was 10.45 years (SD: ± 4.61 years; n = 99) and 47% were male (n = 47). The mean age at the time of AD onset was 1.62 years (SD: ± 2.3 years; n = 99). Age at the time of onset was not affected by sex, familial predisposition for AD, presence of an allergy, use of prednisolone, or maximal strength of topical corticosteroid (*p* < .05 in all analyses).

**Table 1:** Patient characteristics of cohort of 99 eligible cases

	n (%) or mean ± standard deviation (N = 99)
**Male sex**	47 (47.5)
**Age of AD onset, y** **Age at first-line treatment initiation**	1.6 ± 2.3 10.5 ± 4.6
**Use of prednisolone**	28 (28.3)
**Maximum used topical corticosteroid** **Group III** **Group IV** **Allergy type I** **Allergy type IV** **Mother with AD** **Father with AD** **At least one sibling with AD**	53 (53.5) 46 (46.5) 78 (78.6) 18 (18.2) 30 (30.3) 22 (22.2) 28 (28.3)

AD, atopic dermatitis

Among the 99 patients, 63 received MTX as first-line treatment, 32 received AZA, and 4 received CsA. No patients age <18 years received dupilumab within the studied time span. Details on the treatment characteristics are provided in Table 2. Dosing regimens were based on weight, and the lowest recommended dosage was used as the initial dose, except for CsA where the maximum recommended dose was used for immediate effect. In cases of limited effect, dosage was increased within the recommendation based on weight. In the case of CsA, the efficient effect dosage was lowered. Mean age at treatment initiation was 9.6 years (SD: ± 4.2 years) for MTX, 12.0 years (SD: ± 3.4 years) for AZA, and 13.6 years (SD: ± 7.3 years) for CsA (*p* = .02).

**Table 2:** Treatment characteristics of cohort of 99 cases, showing numbers for each individual immunosuppressive treatment

Treatment	Total, n	Male sex, n (%)	<12 y, n ((%)	Age, y, mean ± SD	Treatment time, y			Start dose, mg, mean ± SD	Max dose, mg, mean ± SD
					Mean ± SD	Min.	Max.		
**First-line treatment**									
**MTX**	63	27 (43)	39 (62)	9.6 ± 4.2	1.5 ± 1.2	0.05	4.7	8.2 ± 3.8	10.4 ± 6.4
**AZA**	32	19 (59)	16 (50)	12.0 ± 3.4	2.2 ± 2.3	0.09	8.6	37.5 ± 12.2	89 ± 53.5
**CsA**	4	1 (25)	1 (25)	13.6 ± 7.3	0.8 ± 0.8	0.02	1.7	200 ± 108	222 ± 108
**Second-line treatment**									
**MTX**	12	6 (50)	5 (42)	12.6 ±4.3	1.9 ±1.7	0.05	5.2	9.8±3.8	12.1 ± 4.0
**AZA**	5	3 (60)	4 (80)	10.8 ± 3.9	1.3 ± 1.5	0.2	2.3	35 ± 13.7	55 ± 27.4
**CsA**	7	2 (29)	5 (71)	10.1 ± 4.6	1.2 ± 0.9	0.4	2.3	93.9 ± 51.1	142.8 ± 70.6
**MMF**	2	0 (0)	0 (0)	15.6 ± 2.5	0.3 ± 0.1	0.2	0.3	1500 ± 707	2000

AZA, azathioprine; CsA, cyclosporine; Max, maximum; min, minimum; MMF, mycophenolate mofetil; MTX, methotrexate; SD, standard deviation

Due to small sample sizes for patients with third-and fourth-line treatments, only data for first- and second-line treatments are presented. Data on age refer to age (y) at treatment initiation.

**Fig. 2:**
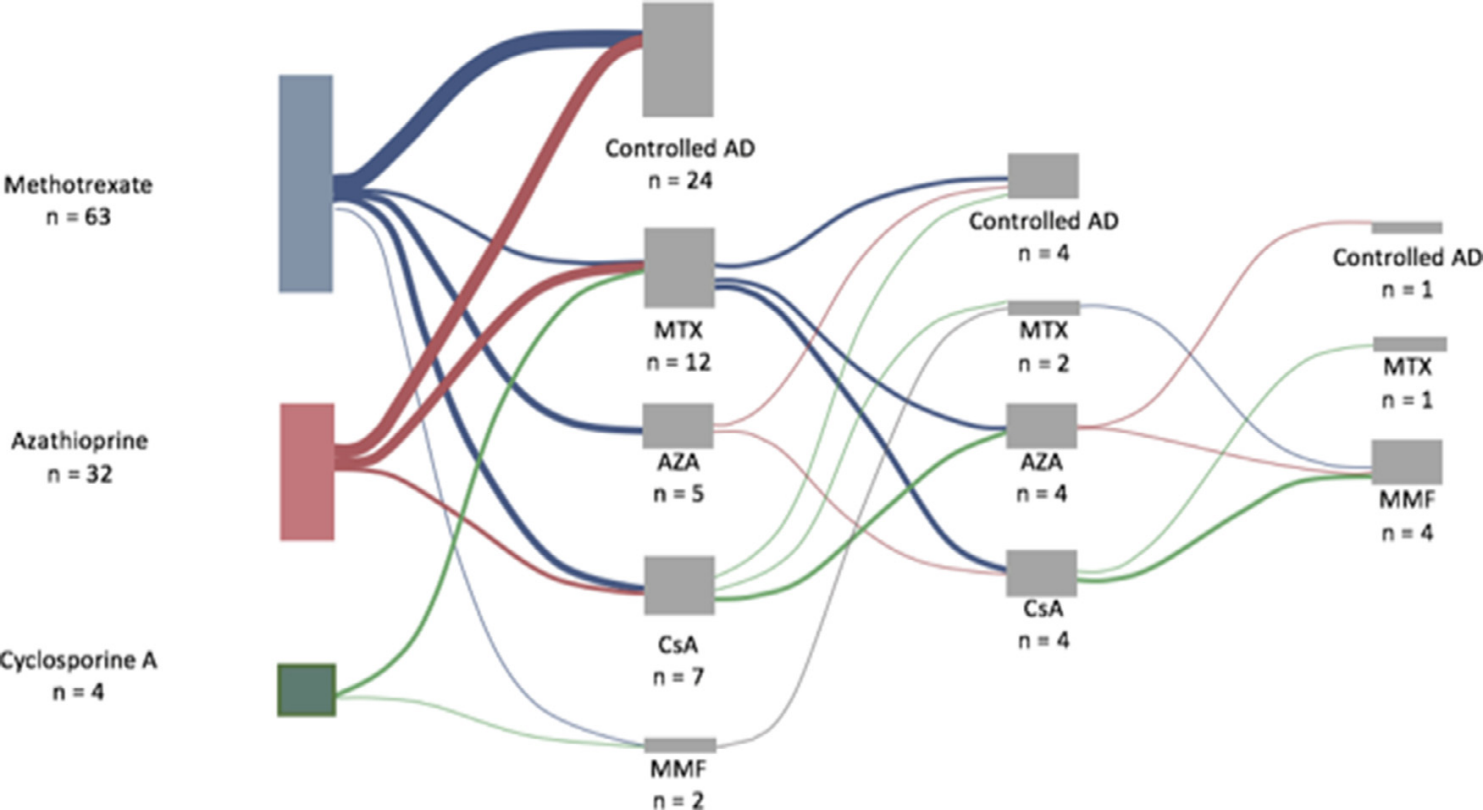
Sankey diagram illustrating the sequence of treatments. The columns of treatment boxes represent the first, second, third, and fourth line of treatment. The size of the box and width of the lines are proportional to the number of patients.

Second-line treatment was initiated in 26 patients, of whom 12 received MTX, 5 received AZA, 7 received CsA, and 2 received MMF. The third-line cohort consisted of 10 patients (MTX: n = 2; AZA: n = 4; CsA: n = 4), and the fourth-line cohort consisted of 5 patients (MTX: n = 1; MMF: n = 4). Figure 2 summarizes the sequences of systemic immunosuppressive treatments the patients received. The cohorts consisting of patients receiving a third- and fourth-line treatment were too small (n < 11), which made extracting valuable information from these treatment lines difficult; thus, they are omitted from the tables and the analysis. Of note, a few patients discontinued MTX, only to resume the same treatment due to relapse in the condition (Fig. 2).

### Treatment effectiveness

Sixty percent of patients receiving MTX as first-line treatment experienced a good effect at the time of discontinuation or data-lock. For AZA as first-line treatment, 53% experienced a good effect. There was no significant difference in treatment effect (good vs. other) across the three first-line treatments (*p* = .344). Among patients receiving MTX as second-line treatment, 25% experienced a good effect. All five patients receiving AZA as second-line treatment had a good effect. The effect of the first-line treatments overall was assessed additionally by examining sleep problems before and during treatment. Information regarding sleep was only available in a subset of patients (n = 46; 46.5%). Of the patients with sleep problems before treatment, 19 recovered during treatment and only four were still having sleep problems. The effect of treatment in relation to an improvement in sleep was highly significant (*p* < .001).

### Reasons for discontinuation

**Fig. 3:**
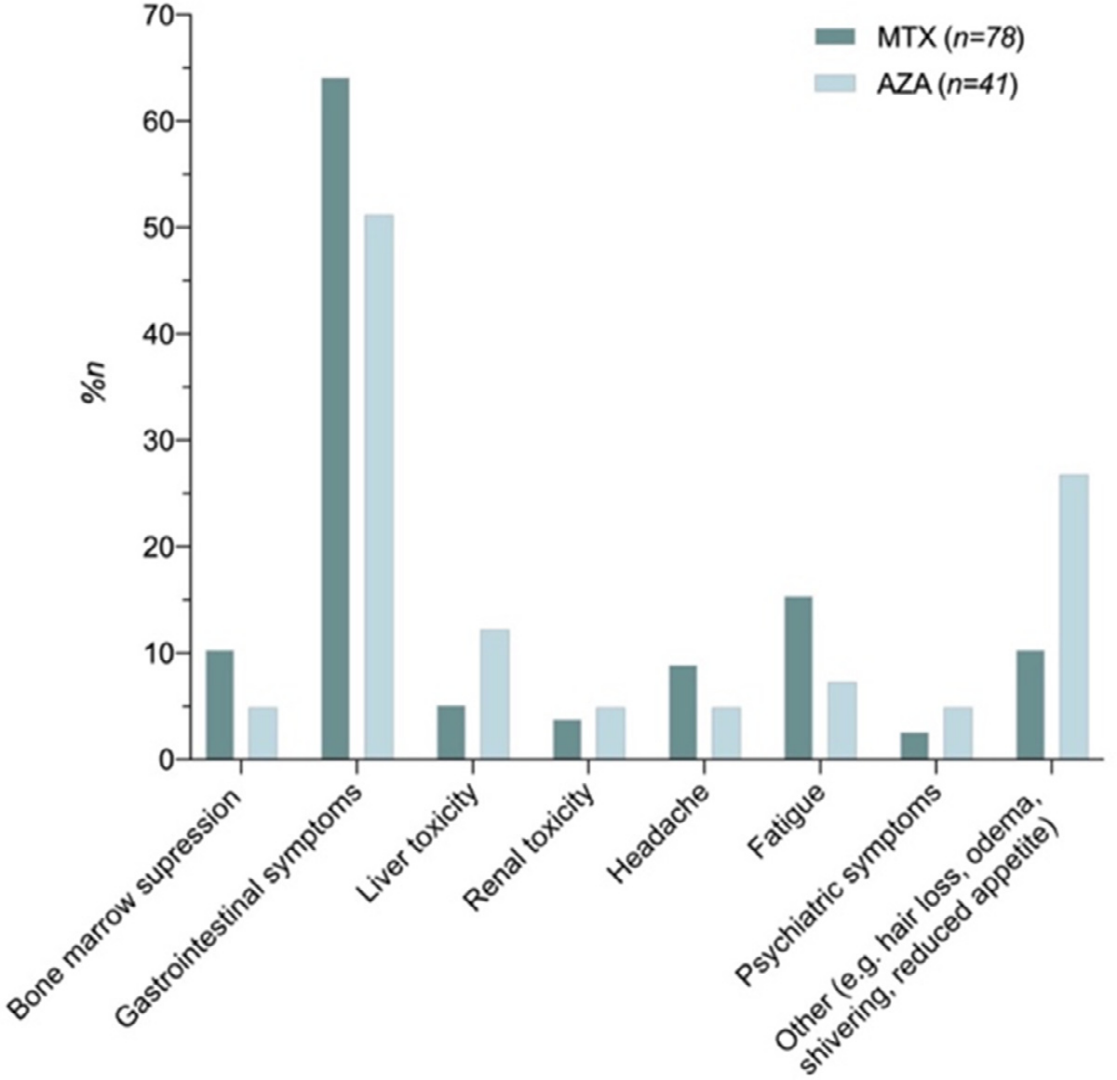
Reported side effects for methotrexate and azathioprine regardless of treatment line, indicated as percent of patients receiving the treatment in one of the treatment lines (i.e., n = 78 for methotrexate; n = 41 for azathioprine).

Discontinuation was analyzed for the first-line treatments (Table 3). There was no difference in reasons for discontinuation (controlled AD vs. other reasons) among MTX, AZA, and CsA (*p* = .42). The same analysis was conducted for the second-line treatments, with the same result (*p* = .786). The main reason for discontinuation was adverse effects for both MTX (25.4%) and AZA (40.6%). Controlled AD was the second most frequent reason for discontinuation for MTX (20.6%) and AZA (34.4%). The total number of adverse effects was 94 for MTX and 48 for AZA regardless of treatment line. The most commonly reported adverse effects were gastrointestinal symptoms. All registered adverse effects for MTX and AZA are presented in Figure 3.

**Table 3:** Reasons for discontinuation of first-line immunosuppressive treatment

Reasons	Methotrexate, n (%)n = 63	Azathioprine, n (%)n = 32	Cyclosporine, n (%)n = 4	*p*-value*
**Controlled atopic dermatitis**	13 (20)	11 (34)	0 (0)	.42
**Inefficacy**	6 (10)	6 (19)	1 (25)	
**Adverse effects**				
**Subjective**	13 (21)	12 (38)	2 (50)	
**Objective**	3 (5)	1 (3)	0 (0)	
**Unknown**	6 (10)	1 (3)	2 (50)	
**Not discontinued**	24 (38)	2 (6)	0 (0)	

⁎ Controlled atopic dermatitis versus inefficacy and adverse effects (without unknown and not discontinued). The sum of the columns exceeds the total number of patients because four patients had an event in two subgroups.

### Drug survival

**Fig. 4:**
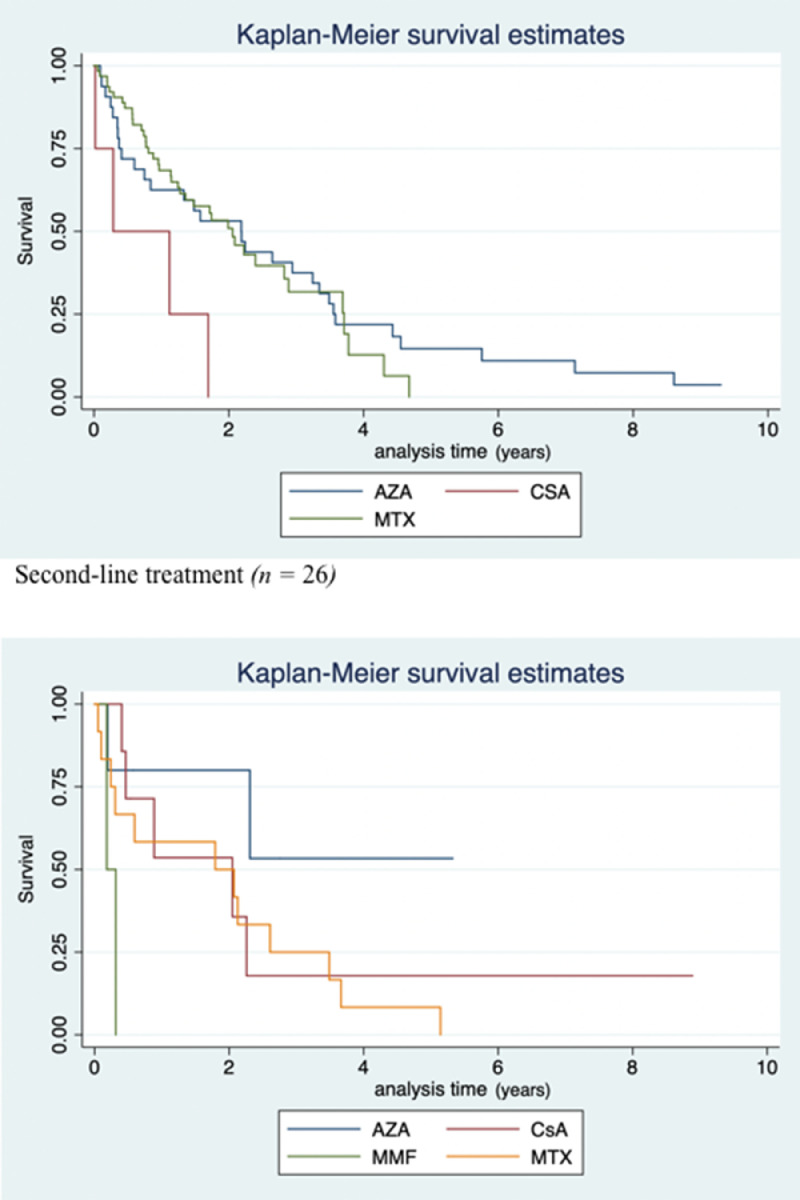
Kaplan–Meier curves of drug survival for first-line (azathioprine: n = 63; methotrexate: n = 32; cyclosporine: n = 4) and second-line (azathioprine: n = 5; methotrexate: n = 12; cyclosporine: n = 7; MMF: n = 2) treatments. The event was discontinuation of treatment. Patients who still received treatment were censored.

The comparison of drug survival for the first-line (AZA, MTX, and CsA) and second-line (AZA, MTX, CsA, and MMF) treatments using Kaplan–Meier curves is presented in Figure 4 The median drug survival for the first-line treatments were 1.58, 1.14, and 0.28 years for AZA, MTX, and CsA, respectively. The drug survival rates for AZA demonstrated that 63%, 53%, and 21% of patients were still receiving treatment after 1, 2, and 4 years, respectively. For MTX, these rates were 69%, 50% and 18%, respectively. The drug survival for CsA was 50% after 1 year. The survival curves for MTX and AZA (as first-line treatment) showed no particular difference; the log-rank test for equality of the survivor functions resulted in a *p*-value of .072, suggesting that the survival functions were equal (on a conventional 0.05% significance level).

The drug survival for patients younger and older than 12 years, for each first-line treatment, was also estimated, but no significant differences were found between the two age groups (hazard ratio: 1.03, 1.07, and 1.08 for MTX, AZA, and CsA, respectively). Age, gender, and drug were examined as determinants of drug survival using a univariate Cox regression analysis. These analyses showed no associations between drug survival and age at initiation or gender. Treatment with AZA in comparison with MTX was a nearly significant predictive factor for longer survival time (hazard ratio: 0.60; 95% confidence interval, 0.36–1.01; *p* = .056)*.* For the second-line treatments, the median survival times were 0.196, 1.794, 0.885, and 0.181 years for AZA, MTX, CsA, and MMF, respectively. One year after treatment initiation, 50% of patients were still receiving MTX, 60% AZA, and 67% CsA.

## Discussion

This drug survival analysis showed that MTX, AZA, and CsA all seemed to be safe and effective in pediatric patients with AD. The median drug survival durations were 1.14, 1.58, and 0.28 years for MTX, AZA, and CsA, respectively. MTX was the most used drug for first-line treatment (n = 63), followed by AZA (n = 32) and CsA (n = 4). The results regarding second- to fourth-line treatment consisted of small patient samples and do not contribute with robust information. Likewise, only a small cohort received CsA as first-line treatment; this reflects the fact that CsA is not used as frequently as MTX and AZA in Denmark (Andersen et al., 2019), most likely owing to a more severe side effect profile, and its limited long-term use.

The choice of first-line therapy, however, varies greatly across Europe. Until recently, when dupilumab was registered, CsA was the only drug licensed for the treatment of AD, and in a questionnaire completed by dermatologists in Europe, CsA was found to be the preferred first‐line systemic therapy for adults in a majority of countries. Other countries preferred MTX, whereas AZA was more frequently used as second-line systemic therapy and rarely as first-line therapy (Vermeulen et al., 2020). Due to the small cohort of patients who received CsA as a first-line treatment, this study mainly contributes with results concerning the use of MTX and AZA.

The results show that MTX and AZA had similar survival curves. The drug survival rates for MTX were 69%, 50%, and 21% after 1, 2, and 4 years, respectively. For AZA, these rates were 63%, 53%, and 18%, respectively. Similarly, the median drug survival duration did not vary greatly among these treatments. Of note, drug survival is affected by the occurrence of spontaneous recovery, which might happen naturally when children grow older. The primary reasons for discontinuation for both MTX and AZA were adverse effects (MTX: 25.4%; AZA: 40.6%), followed by controlled AD (MTX: 20.6%; AZA: 34.3%). The adverse effects were mainly gastrointestinal symptoms for both MTX and AZA (Fig. 3).

Prior drug survival studies (in adults) found results that differ from ours, but many factors may be responsible for these variations. Politiek et al. (2016) found drug survival rates for MTX of 41% after 1 year and 34% after 2 years with a median drug survival time of 9.8 months, demonstrating a shorter drug survival for MTX. On the other hand, Gerbens et al. (2018) found drug survival rates for MTX slightly higher than ours, with rates of 76% after 1 year and 53% after 2 years and a median drug survival time of 28.8 months. For AZA, both Gerbens et al. (2018) and Van der Shaft et al. (2016) demonstrated shorter drug survival in their study, with median drug survival times of 11.5 and 6 months, respectively.

These differences might be the result of the study designs, physician preferences, adherence to treatment, or disease severity. Furthermore, spontaneous recovery is higher in pediatric patients, contributing to a shorter drug survival. In addition, drug survival may be influenced by patients’ expectations and satisfaction with the treatment, and whether other treatment options are available. Discontinuation due to adverse effects showed considerably high prevalence in our study, being the main reason for discontinuation for both MTX and AZA. This might be because the cohort consisted of children, in whom adverse effects are less acceptable than in adult patients. Another factor that might contribute is that the treatments were first-line choices, and other recognized treatment options were available. Thus, in the event of any adverse effect, it might be tempting to change treatment course rather than accept adverse effects.

In addition to drug survival, the present study also examined the effect of treatment. The results showed that the majority of patients on both MTX (60%) and AZA (53%) experienced a good effect of the treatment at the time of discontinuation or data-lock. There was no significant difference in treatment effect between MTX and AZA.

An important finding of this study was that patients on either immunosuppressive treatment showed a highly significant improvement in sleep quality, posing an important quantitative marker for the effect of the treatments. Considering that sleep problems are an important marker for disease severity in children and that sleep problems can greatly affect the quality of life of patients with AD, the finding is encouraging and speaks to the validation of the use of immunosuppressive drugs in treating severe AD in children. These findings suggest that MTX and AZA are very valuable treatment options in children. However, it must be noted that the findings regarding sleep quality were available only in a subset of patients (n = 46 of 99), which raises the possibility that the result may be biased.

Overall, our results provide evidence for the use of MTX and AZA in children with severe AD and indicate that these treatments are equal in both drug survival, reason for discontinuation, and treatment effect. Discontinuation due to adverse effects was slightly higher for AZA (41%) compared with MTX (26%); however, this was not statistically significant. Thus, the treatments are equally suitable in the treatment of pediatric patients, making the choice between the two a question of preference and individual differences in tolerability. There may be differences concerning long-term adverse effects regarding a small increased risk of cancer in patients treated with AZA, which makes long-term treatment with MTX more favorable.

The retrospective study design and the large amount of subjective data pose a potential risk of information bias. However, all patients were followed and treated in the same dermatology department by the same group of doctors, probably limiting this risk. In terms of strengths, the study consists of a relatively large study population, strengthening the external validity of the results.

Future work is necessary to gain a dependable evaluation of the daily practice results of using immunosuppressive therapies in children. In addition, this study mainly contributes with results concerning MTX and AZA; thus, there remains a knowledge gap regarding the drug survival of CsA and MMF in pediatric cohorts. Furthermore, many future biologic treatments are emerging and may be in competition with conventional immunosuppressive treatments. Still, to guide the use of new treatments, it is imperative that the properties of the currently available treatments be described in more detail.

## Conclusion

This study presents the first drug survival analysis of immunosuppressive treatments in a cohort of pediatric patients with severe AD. The immunosuppressive treatments MTX and AZA showed similar drug survival and efficacy and must be considered equally valuable treatments. Furthermore, both treatments contributed to highly significant improvement in patients’ sleep quality. As the prevalence of AD among children has been increasing and new therapies are emerging, these results may guide clinical decision-making in the treatment of severe AD in children.

## Funding

None.

## Study approval

N/A

## Conflicts of interest

None.
